# Advanced imaging characterization of post-chemoradiation glioblastoma stratified by diffusion MRI phenotypes known to predict favorable anti-VEGF response

**DOI:** 10.1007/s11060-025-05019-8

**Published:** 2025-04-14

**Authors:** Francesco Sanvito, Irina Kryukov, Jingwen Yao, Ashley Teraishi, Catalina Raymond, John Gao, Cole Miller, Phioanh L. Nghiemphu, Albert Lai, Linda M. Liau, Kunal Patel, Richard G. Everson, Blaine S.C. Eldred, Robert M. Prins, David A. Nathanson, Noriko Salamon, Timothy F. Cloughesy, Benjamin M. Ellingson

**Affiliations:** 1 UCLA Brain Tumor Imaging Laboratory (BTIL), Center for Computer Vision and Imaging Biomarkers, University of California Los Angeles, Los Angeles, CA USA; 2 Department of Radiological Sciences, David Geffen School of Medicine, University of California Los Angeles, Los Angeles, CA USA; 3 Department of Neurology, David Geffen School of Medicine, University of California Los Angeles, Los Angeles, CA USA; 4 UCLA Neuro-Oncology Program, David Geffen School of Medicine, University of California Los Angeles, Los Angeles, CA USA; 5 Department of Neurosurgery, David Geffen School of Medicine, University of California, Los Angeles, Los Angeles, CA USA; 6 Department of Pharmacology, David Geffen School of Medicine, University of California Los Angeles, Los Angeles, CA USA; 7 Department of Bioengineering, Henry Samueli School of Engineering and Applied Science, University of California Los Angeles, Los Angeles, CA USA; 8 Medical Scientist Training Program, David Geffen School of Medicine, University of California, Los Angeles, Los Angeles, CA USA; 9 Department of Psychiatry and Biobehavioral Sciences, David Geffen School of Medicine, University of California, Los Angeles, Los Angeles, CA USA

**Keywords:** Glioblastoma, Diffusion-weighted imaging, Perfusion-weighted imaging, Anti-VEGF, Anti-angiogenic, Bevacizumab

## Abstract

**Purpose:**

Recurrent glioblastomas showing a survival benefit from anti-VEGF agents are known to exhibit a distinct diffusion MRI phenotype. We aim to characterize advanced imaging features of this glioblastoma subset.

**Methods:**

MRI scans from 87 patients with IDH-wildtype glioblastoma were analyzed. All patients had completed standard chemoradiation and were anti-VEGF-naïve. Contrast-enhancing tumor segmentations were used to extract: the lowest peak of the double gaussian distribution of apparent diffusion coefficient values (ADC_L_) calculated from diffusion MRI, relative cerebral blood flow (rCBV) values from perfusion MRI, MTR_asym_ @ 3ppm from pH-weighted amine CEST MRI, quantitative T_2_ and T_2_* relaxation times (qT_2_ and qT_2_*), T_1_w subtraction map values, and contrast-enhancing tumor volume. Lesions were categorized as high- or low-ADC_L_ using a cutoff of 1240 µm^2^/s, according to previous studies.

**Results:**

High-ADC_L_ lesions showed significantly lower rCBV (1.02 vs. 1.28, *p* = 0.0057), higher MTR_asym_ @ 3ppm (2.36% vs. 2.10%, *p* = 0.0043), and higher qT_2_ (114.8 ms vs. 100.9 ms, *p* = 0.0094), compared to low-ADC_L_ lesions. No group differences were seen in contrast-enhancing tumor volume, T_1_w subtraction map values, and qT_2_*, nor in clinical variables such as sex category, MGMT status, and EGFR status. Finally, no clear group-specific preferential locations were seen.

**Conclusion:**

Post-chemoradiation glioblastomas with a diffusion MRI phenotype that is known to predict a favorable response to anti-VEGF (ADC_L_ ≥1240 µm^2^/s) have distinct biological features, with different perfusion and metabolic characteristics, and T_2_ relaxation times.

**Supplementary Information:**

The online version contains supplementary material available at 10.1007/s11060-025-05019-8.

## Introduction

Glioblastoma is the most frequent primary brain tumor, and bears very poor prognosis, with only an estimated 5–6% survival rate at 5 years in the general population [1]. VEGF signaling plays a central role in glioblastoma biology by promoting neoangiogenesis [2, 3]. Despite the initial enthusiasm for the opportunity of using anti-VEGF agents to treat this disease, randomized phase III clinical trials conducted in the last decade failed to demonstrate an overall survival (OS) advantage from the administration of anti-VEGF agents, either in the newly-diagnosed [4, 5] or recurrent [6] setting. However, some evidence suggests that certain patient subsets, whose lesions exhibit specific diffusion MRI characteristics, may benefit from anti-VEGF therapies [7-14]. Diffusion MRI evaluates the Brownian motion of water molecules within the tissue [15] and can be used to estimate an apparent diffusion coefficient (ADC). Measures of ADC have been shown to correlate with histological cell density [16-23] and may also reflect other microstructural features of the tissue, such as composition of the extracellular matrix [10, 24, 25]. Results from multiple independent cohorts showed that ADC-low (ADC_L_), the mean value of the lower peak of a double-gaussian mixed model fit to the ADC histogram extracted from the contrast-enhancing tumor regions, is a predictor of survival in patients with recurrent glioblastomas treated with a variety of anti-VEGF agents [7-14]. These findings suggest that, while anti-VEGF therapies did not historically show an OS benefit in phase III trials, a specific subset of patients with recurrent glioblastoma exhibiting this peculiar diffusion MRI phenotype may actually have a survival benefit with anti-VEGF agents. Notably, the ADC_L_ cutoff value predictive of survival was highly consistent across independent cohorts [7-10, 13, 14]. Despite evidence suggesting that diffusion MRI may be predictive of response to anti-VEGF therapy, the potential association with other imaging measurements remains unknown, and a comprehensive imaging characterization of these recurrent glioblastoma subsets is still lacking.

Overall, these studies suggest that there may be inherent biological differences in different subgroups of glioblastomas, possibly arising after the standard first-line chemoradiation. In the current study we examined advanced imaging characteristics of glioblastomas imaged after completion of chemoradiation (“post-chemoradiation glioblastomas”), stratified by ADC_L_. We theorize that diffusion phenotypes may correspond to distinct advanced imaging profiles with respect to vascularity, acidity, T_2_ and T_2_* relaxation time, contrast-enhancement intensity, localization, and extent of tumor burden.

## Materials and methods

### Patient selection

Brain lesions imaged at our institution between May 2017 and December 2021 were retrospectively reviewed. Inclusion criteria were as follows: (1) received a histological diagnosis of IDH-wildtype glioblastoma; (2) the images were acquired after surgical resection and after the completion of concurrent chemoradiation; (3) did *not* previously receive anti-VEGF therapy, because this treatment is known to impact diffusion MRI metrics [26, 27] and because previous literature showed the predictive role of ADC_L_ phenotypes in *pre*-anti-VEGF MRI scans [7-10]; (4) exhibits a RANO-defined measurable contrast-enhancing (CE) tumor component as measured on 3D imaging (≥ 1 cm x ≥ 1 cm x ≥ 1 cm) [28], on the MRI scan used for the analysis. For each patient, only the most recent MRI meeting the inclusion criteria was analyzed. Patients gave written informed consent to be included in the database used in this study, which was approved by the institutional IRB (IRB#19-002084).

### Imaging acquisition and analysis

MRI images were acquired at 3T on Siemens scanners (Magnetom Prisma, Skyra, or Trio), with a standardized brain tumor imaging protocol (BTIP) [29, 30]. MRI sequences included parameter-matched 3D T_1_-weighted images before (T_1_w) and after (CE-T_1_w) injection of contrast agent, diffusion-weighted imaging (DWI), dynamic susceptibility contrast (DSC) perfusion imaging, and pH-weighted amine chemical exchange saturation transfer (CEST) either with single-echo echo-planar imaging (EPI) or with spin-and-gradient-echo EPI (SAGE-EPI). DWI images were acquired with *b*-value = 0 and *b*-value = 1000 s/mm² and were used to calculate the apparent diffusion coefficient (ADC). DSC images were motion corrected, and relative cerebral blood volume (rCBV) was calculated after bidirectional leakage correction [31] and normalized to the median rCBV of the brain, as proposed in previous studies [32].

Amine CEST sequence consisted of three 100 ms Gaussian-shaped saturation pulses, each with a peak amplitude of 6 µT, and with a 5 ms delay between pulses. After the pulse train, spoiling gradients were applied before the single-shot gradient-echo EPI readout. Z-spectral points were sampled heavily around + 3.0 ppm, -3.0 ppm, and 0 ppm, corresponding to amine proton resonance frequency, reference frequency, and water resonance frequency, respectively [33, 34]. Magnetic transfer ratio asymmetry at 3 ppm (MTR_asym_ @ 3ppm) was computed using in-house MATLAB code (Mathwoks, version R2023b) through: (1) motion correction of CEST using the *mcflirt* function from FSL (FMRIB software library; University of Oxford; https://fsl.fmrib.ox.ac.uk/fsl/); (2) B_0_ inhomogeneity correction through a z-spectra-based k-means clustering and Lorentzian fitting algorithm [35]; (3) calculation of MTR_asym_ at amine proton resonance frequency (3.0ppm) integrated over a 0.4ppm range [33]. MTR_asym_ @ 3ppm and ΔB_0_ maps were evaluated to exclude from analysis cases with excessive motion artifacts or excessing static magnetic field inhomogeneity (ΔB_0_ > 0.3 ppm) in the tumor area [35]. In a subset of patients for whom CEST images were acquired through a spin-and-gradient echo EPI (CEST-SAGE-EPI), quantitative T_2_ relaxation times (qT_2_) [ms] and T_2_* relaxation times qT_2_* [ms] were computed by applying the Bloch equations to the CEST-SAGE-EPI S_0_ images (i.e., reference images with the same sequence parameters as CEST-SAGE-EPI but without saturation pulse), which composed of two gradient echoes, one mixed echo, and one spin echo [33].

T_1_w, ADC, rCBV, MTR_asym_ @ 3ppm, qT_2_, and qT_2_* maps were registered to CE-T_1_ with a linear registration, using the built-in FSL *flirt* function. After co-registration, skull stripping and intensity normalization, CE-T_1_w and T_1_w images were subtracted voxel-wise to generate T_1_w subtraction maps (deltaT_1_) [36]. DeltaT_1_ values reflect the degree of contrast enhancement, and can be considered a semiquantitative measure of blood-brain barrier permeability. A 3D segmentation of the contrast-enhancing volume of the tumor was created automatically using the NS-HGlio v.2.0 deep learning algorithm (Neosoma Inc, https://neosomainc.com/) [37]. Registrations and segmentations were quality checked, and manual adjustments were performed when needed.

The tumor segmentation was used to extract the distribution of ADC values, and a double gaussian fitting was applied to ADC histograms. The ADC-low (ADC_L_) value corresponded to the mean of the gaussian distribution with the lowest ADC values, as previously described [7-10]. Lesions were categorized based on their ADC_L_ into high-ADC_L_ group (ADC_L_ ≥1240 µm^2^/s) and low-ADC_L_ group (ADC_L_ <1240 µm^2^/s). The ADC_L_ cutoff of 1240 µm^2^/s was previously validated as predictive for anti-VEGF therapy success on multiple independent cohorts [7, 8, 10, 13, 14], and was shown to be robust when applied to datasets acquired with different protocols, using different scanners, at either 1.5 or 3.0 T [7]. Contrast-enhancing tumor segmentations were also used to extract median rCBV, median MTR_asym_ @ 3ppm, median qT_2_, median qT_2_*, and median deltaT_1_ values.

Lesion locations were categorized as either frontal, temporal, parietal, occipital, deep-seated supratentorial, or infratentorial. In addition, CE-T_1_w images were registered to the MNI 1 mm isotropic normalized brain with the built-in FSL *flirt* function, and the resulting registration matrix was applied to contrast-enhancing tumor segmentations to generate frequency maps of high-ADC_L_ and low-ADC_L_ lesions, as in previous studies [38, 39].

Finally, since corticosteroid administration can influence ADC values [40], the corticosteroid daily dose (in dexamethasone equivalent dose) administered at the time of the MRI scan was annotated.

### Statistical analysis

Statistical analyses were performed using GraphPad Prism (version 8.4.3). Since not all group observations passed the Shapiro-Wilk normality test, a Mann-Whitney *U*-test was used to evaluate group differences in contrast-enhancing tumor volume, median deltaT_1_, median rCBV, median MTR_asym_ @ 3ppm, median qT_2_, median qT_2_*, and corticosteroid dosage between high- and low-ADC_L_ groups. Since amine CEST MTR_asym_ @ 3ppm can be influenced by tissue T_2_ [41], a multivariate linear regression was conducted to test whether MTR_asym_ @ 3ppm median values were significantly different between groups, while adjusting for qT_2_ values. Associations between MRI metrics were tested with Pearson’s correlations. Fisher’s exact tests were conducted to evaluate differences between high- and low-ADC_L_ groups in terms of lesion location, sex category, MGMT methylation, and EGFR amplification. Statistical significance was set to *p* < 0.05 and Benjamini-Hochberg corrections were performed in case of multiple comparisons.

To better visualize how multiparametric imaging measurements group between diffusion MR phenotypes, *k*-means clustering with *k* = 2 clusters was performed using ADC_L_ (used as a continuous measure, not dichotomized) and advanced MRI metrics showing a significant difference between high- and low-ADC_L_ groups. *k*-means clustering was performed using in-house code based on the Python *sklearn* library.

## Results

### Patient selection

**Fig. 1:**
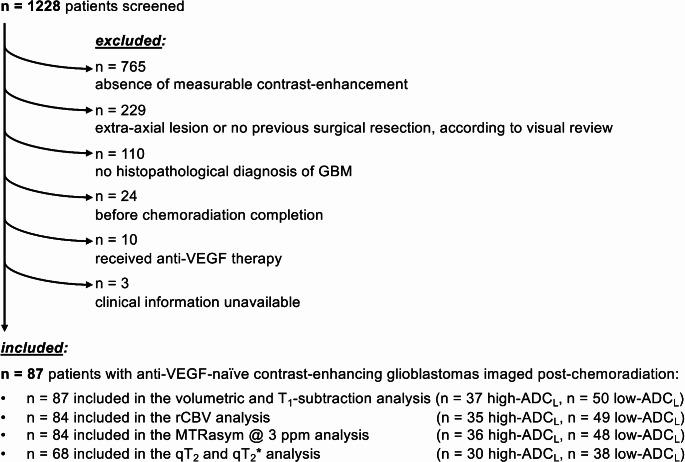
Flow-chart of patients included and excluded from the study

MRI scans and clinical information from 1228 patients were initially screened. Based on the inclusion and exclusion criteria, 87 patients were selected for the analyses (Fig. 1; Table 1). Out of these 87 patients, 37 exhibited ADC_L_ ≥1240 µm^2^/s (high-ADC_L_) and 50 had ADC_L_ <1240 µm^2^/s (low-ADC_L_) within areas of contrast-enhancing tumor. All 87 cases were included in the analysis of tumor locations, contrast-enhancing tumor volume, and T_1_-subtraction values. 84 patients were included in the rCBV analysis, while 3 patients were excluded due to the presence of intralesional blood products preventing a reliable rCBV estimation. 84 patients were included in the MTR_asym_ @ 3ppm analysis, while 3 were excluded due to excessive motion artifacts or high static magnetic field inhomogeneity involving the contrast-enhancing tumor region. 68 patients had multi-echo SAGE-EPI data and were included in the qT_2_ and qT_2_* analysis.

**Table 1:** Descriptive characteristics of the patient cohort

Characteristic	High-ADC_L_ group (ADC_L_ ≥1240, *n* = 37)	Low-ADC_L_ group (ADC_L_ <1240, *n* = 50)
Median age (IQR)	60 years (12)	60 years (15)
Sex, n (%)		
Male	25 (67.6%)	28 (56.0%)
Female	12 (32.4%)	22 (44.0%)
MGMT status, n (%)		
Methylated	13 (35.1%)	19 (38.0%)
Unmethylated	20 (54.1%)	30 (60.0%)
Unknown	4 (10.8%)	1 (2.0%)
EGFR amplification, n (%)		
Amplified	16 (43.2%)	25 (50.0%)
Non-amplified	15 (40.6%)	21 (42.0%)
Unknown	6 (16.2%)	4 (8.0%)
Contrast-enhancing volume (IQR)	21.4 cc (21.1)	16.4 cc (23.7)
Location of lesion, n (%)		
Frontal	7 (18.9%)	22 (44.0%)
Temporal	13 (35.1%)	8 (16.0%)
Parietal	12 (32.4%)	12 (24.0%)
Occipital	3 (8.1%)	5 (10.0%)
Other	2 (5.4%)	3 (6.0%)

### Clinical characteristics and locations of high-ADC_L_ and low-ADC_L_ tumors

No significant group differences were found in the prevalence of sex category, MGMT methylation, or EGFR amplification (Supplementary Fig. 1). Additionally, no difference in corticosteroid dosage were seen between the two groups, suggesting that the categorization according to MR diffusion phenotypes was not contaminated by corticosteroid dose. The visualization of the frequency maps of high-ADC_L_ and low-ADC_L_ tumors did not reveal any evident group differences in lesion location (Supplementary Fig. 2A–B). When analyzing lesion locations as categorical variables, tumors with high ADC_L_ appeared to be less frequently located in the frontal lobes (*n* = 7 vs. *n* = 22, *p* = 0.0209) and more frequently located in the temporal lobes (*n* = 13 vs. *n* = 8, *p* = 0.0464), compared to lesions with low ADC_L_ (Supplementary Fig. 2C–D). However, these differences were not considered significant after Benjamini-Hochberg correction for false discovery rate. No difference in the frequency of locations within other anatomical regions were observed (Supplementary Fig. 2E–G).

### MRI features of high-ADC_L_ and low-ADC_L_ tumors

**Fig. 2:**
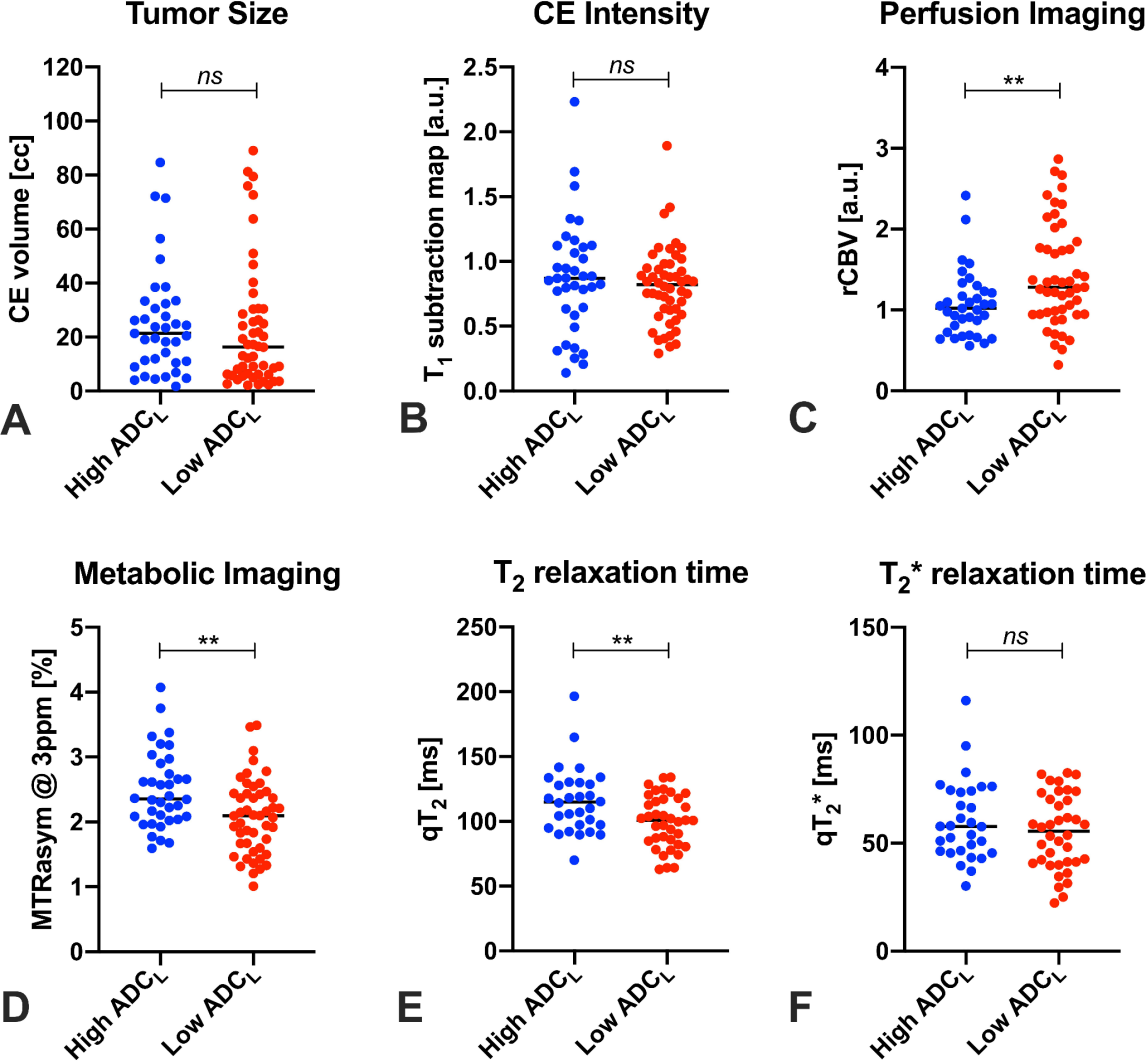
Differences in multiparametric MRI metrics between diffusion MRI phenotypes. Lesions bearing a high-ADC_L_ MR diffusion phenotype (ADC_L_ ≥1240 µm^2^/s) exhibit lower rCBV (**C**), higher MTR_asym_ @ 3ppm (**D**), and higher qT_2_ (**E**) compared to the low-ADC_L_ group. The differences in MTR_asym_ @ 3ppm may be partly due to underlying qT_2_ differences, but multivariate analyses showed significant differences in MTR_asym_ @ 3ppm even after adjusting for qT_2_. No significant group differences were seen in contrast-enhancing tumor volume (**A**), contrast-enhancement intensity (**B**), and qT_2_* (**F**). a.u. = arbitrary units, cc = cubic centimeters, ms = milliseconds. **p < 0.01 surviving a Benjamini-Hochberg correction

No significant differences in contrast-enhancing tumor size (Fig. 2A) or contrast-enhancement intensity (Fig. 2B) were seen between groups. Lesions with high ADC_L_ were found to have a lower median rCBV (1.02 vs. 1.28, *p* = 0.0057, Fig. 2C) and higher median MTR_asym_ @ 3ppm (2.36% vs. 2.10%, *p* = 0.0043, Fig. 2D) compared to lesions with low ADC_L_. Differences in MTR_asym_ @ 3ppm between groups were significant also in multivariate analysis adjusting for qT_2_ (β = 0.60%, *p* < 0.0001, R^2^ = 0.24). High-ADC_L_ lesions also exhibit a longer median qT_2_ compared to lesions with low ADC_L_ (114.8 ms vs. 100.9 ms, *p* = 0.0094, Fig. 2E), while qT_2_* measurements showed no significant differences between groups (Fig. 2F). Measurements of median qT_2_ were correlated with qT_2_*, and with ADC_L_, but not with MTR_asym_ @ 3ppm (Supplementary Fig. 3).

### Representative cases of high-ADC_L_ and low-ADC_L_ tumors

**Fig. 3:**
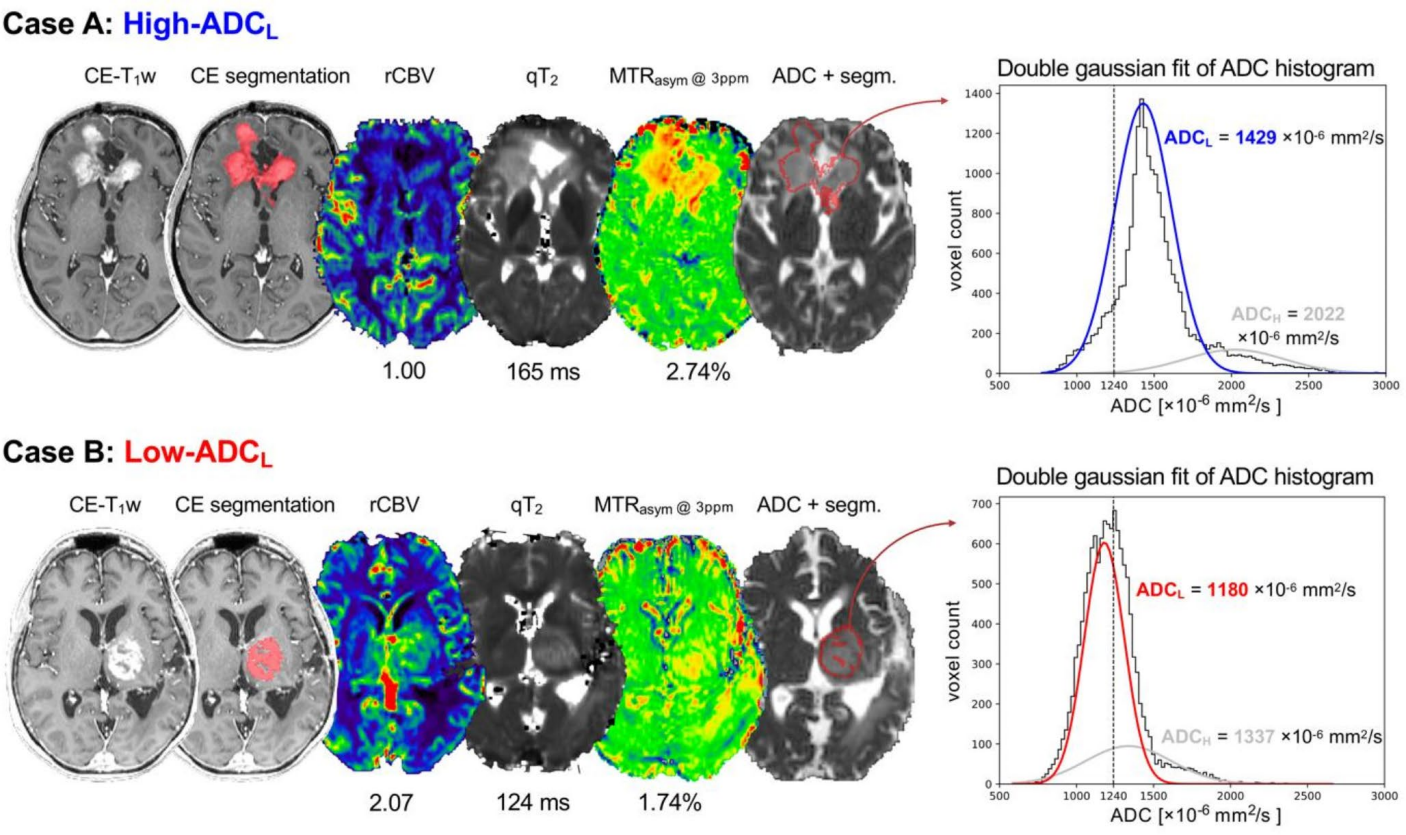
Representative cases. The figure shows post-chemoradiation multiparametric MRI images obtained from a 38-year-old female patient with a frontal glioblastoma WHO grade 4, IDH wild-type, MGMT unmethylated, EGFR non-amplified (case **A**), and from a 61-year-old female patient with a thalamic glioblastoma WHO grade 4, IDH wild-type, MGMT methylated, EGFR non-amplified (case **B**). In addition to higher ADC_L_, case **A** also exhibited lower rCBV, higher MTR_asym_ @ 3ppm, and longer qT_2_, compared to case **B**

Selected representative cases summarize the multiparametric imaging findings in the two groups of post-chemoradiation glioblastomas (Fig. 3). The case of a 38-year-old female patient shows a frontal glioblastoma WHO grade 4, with involvement of the genu of the corpus callosum, MGMT unmethylated, EGFR non-amplified (Fig. 3A). The ADC values are homogeneously relatively high within the contrast-enhancing tumor tissue, and the lesion belongs to the high-ADC_L_ group. The area of contrast-enhancement is also characterized by low rCBV values, relatively high MTR_asym_ @ 3ppm, and long qT_2_ (Fig. 3A). In comparison, the case of a 61-year-old female patient is a paradigmatic example of low-ADC_L_ tumor (Fig. 3B). This thalamic glioblastoma WHO grade 4, MGMT methylated, EGFR non-amplified, shows clear differences compared to the previous case, on multiparametric MRI. In addition to lower values of ADC, the area of contrast-enhancement in the left thalamus is characterized by evident higher rCBV values, lower MTR_asym_ @ 3ppm, and shorter qT_2_ (Fig. 3B).

### Multiparametric k-means clustering of MRI features

The *k*-means clustering analysis was conducted with continuous values of ADC_L_, median rCBV, and median MTR_asym_ @ 3ppm, as the previous group analyses showed that the values of these metrics were significantly different between ADC_L_ groups. While also qT_2_ values were different between groups, they were excluded from the clustering analysis due to their correlation with ADC_L_ values (Supplementary Fig. 3).

**Fig. 4:**
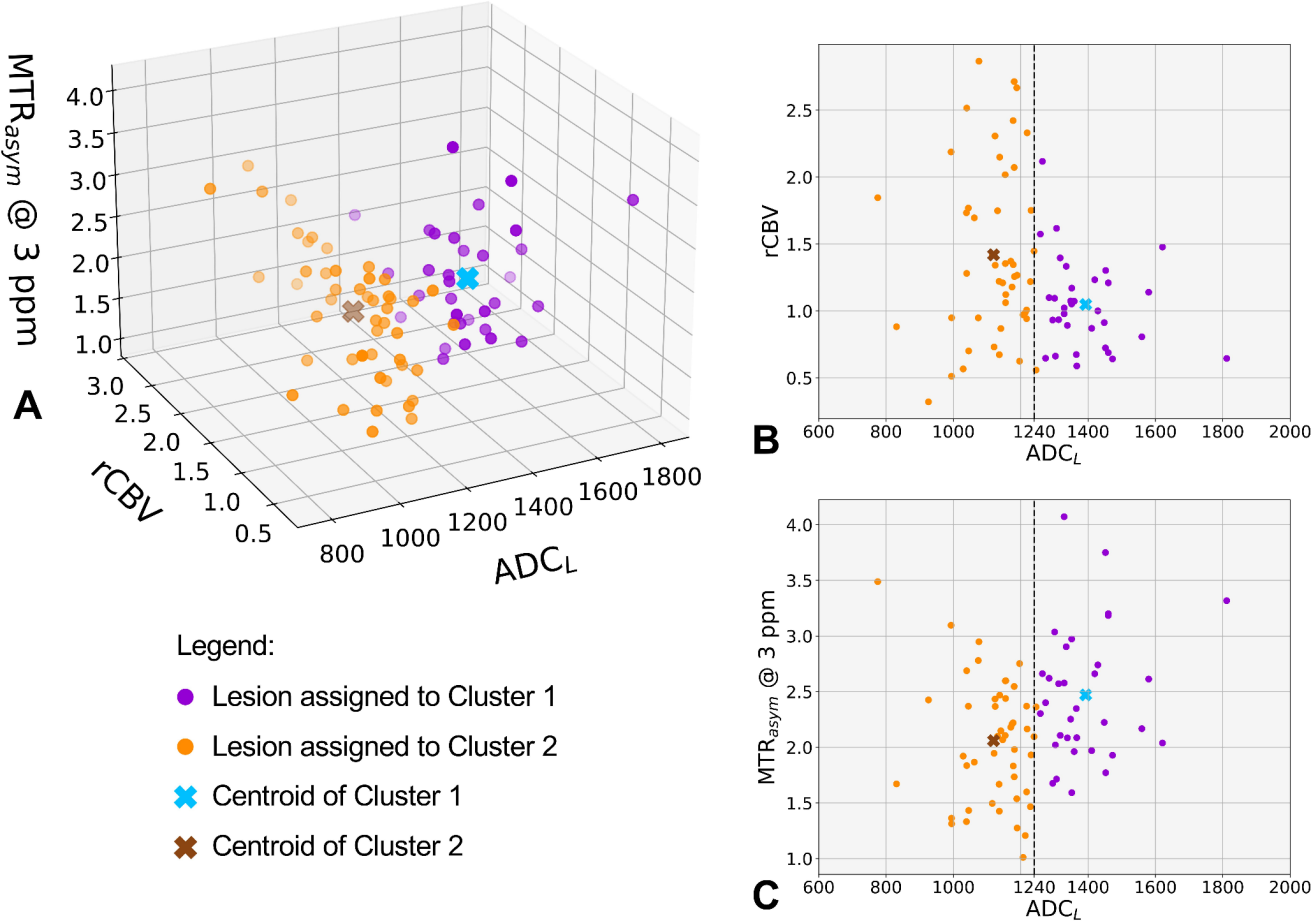
Multiparametric *k*-means clustering of contrast-enhancing tumor quantitative MRI metrics. A *k*-means clustering analysis was conducted using ADC_L_ (as a continuous variable), median rCBV, and median MTR_asym_ @ 3 ppm extracted from the contrast-enhancing tumor. *k*-means identified two distinct clusters (cluster 1 and cluster 2), which almost perfectly corresponded to the high-ADC_L_ and low-ADC_L_ groups, respectively, with the exception of only one high-ADC_L_ lesion assigned to cluster 2

The multiparametric *k*-means clustering showed distinct groups with well-defined diffusion, perfusion, and MTR_asym_ @ 3ppm values (Fig. 4). Cluster 1 (purple) exhibited higher ADC_L_, lower rCBV, and higher MTR_asym_ @ 3ppm compared to cluster 2 (orange). Of note, ADC_L_ was used as a *continuous* variable and had the same weight as rCBV and MTR_asym_ @ 3ppm, in this analysis. Yet, the two clusters showed a “natural” separation based on a ADC_L_ threshold around 1240 µm^2^/s (Fig. 4B–C). Indeed, cluster 1 almost perfectly overlapped with the high-ADC_L_ group, and cluster 2 with the low-ADC_L_ group, with the exception of only *n* = 1 lesion with ADC_L_ 1246 µm^2^/s (high-ADC_L_ group) that was assigned to cluster 2. This suggests that, while these two groups may differ in different biological aspects that can be measured with advanced MRI, an ADC_L_ threshold of 1240 µm^2^/s may represent a sufficient parameter to separate these groups accurately.

## Discussion

The results of this study showed that post-chemoradiation glioblastomas exhibiting distinct diffusion MRI phenotypes predictive of anti-VEGF response also are characterized by peculiar biological features that are reflected in lower rCBV, higher MTR_asym_ @ 3ppm, and longer T_2_ relaxation times (qT_2_).

The observation that lesions that are more likely to obtain a survival advantage from anti-VEGF (high-ADC_L_ lesions) exhibit lower rCBV is consistent with prior studies demonstrating a favorable prognostic value of low rCBV in recurrent glioblastomas treated with anti-VEGF therapy [42-44]. In particular, Kickingereder et al. reported that low rCBV values were predictive of longer survival only in their sub-cohort treated with anti-VEGF agents, and not in their sub-cohort treated with alkylating agents, suggesting that rCBV may be a specific predictor of anti-VEGF efficacy rather than general prognosis [42]. Overall, considering our results as well as the evidence from the literature, lesions showing a survival advantage from anti-VEGF may be characterized by lower rCBV values, indicative of a lower vascular density [45]. This could be interpreted as anti-VEGF agents being potentially more effective in lesions that have a less pronounced neoangiogenesis, while lesions with a more developed vascular network can resist anti-VEGF. This interpretation is also in line with histopathological findings revealing that the residual tumor cells during anti-VEGF treatment are predominantly located around the remaining vasculature (vascular co-option) [46]. Therefore, a more pronounced tumor burden may survive in glioblastomas with more abundant vascular structures. Additionally, other mechanisms may play a role in the development of anti-VEGF resistance, such as “salvage” neoangiogenic pathways alternative to VEGF [47, 48], that may be activated by lesions that resist anti-VEGF treatments and that may also be associated with different perfusion MRI profiles. Despite the evidence cited, however, a positive predictive role of low rCBV for anti-VEGF treatments should not be considered conclusive, since other findings suggest that also lesions with high rCBV may exhibit a survival benefit when treated with anti-VEGF [49]. More in general, many adaptive phenotypical changes take place in glioblastomas treated with anti-VEGF, such as epithelial-mesenchymal transition [48], and the mechanism of anti-VEGF resistance are complex and multifactorial.

In our study, lesions with high ADC_L_ also exhibited higher values of MTR_asym_ @ 3ppm. This finding is in line with a previous study on high-grade gliomas, reporting a positive correlation between ADC values and CEST MRI measurements obtained with a saturation duration of 2 s and a saturation power level of B1 (rms = 2.0 µT), therefore sensitive to amine groups [50]. MTR_asym_ @ 3ppm from amine CEST MRI is a metabolic metric that reflects tissue acidity, in presence of high aminoacidic content and long tissue T_2_ [33, 51]. Higher values of MTR_asym_ @ 3ppm may be the result of a more acidic tumor microenvironment and/or a higher content of amino acids with aminic groups [33, 51]. A potential role of CEST MRI for predicting anti-VEGF treatment outcomes is supported by previous literature showing that changes in amine CEST MRI [52] and amide CEST MRI [53] upon bevacizumab initiation were predictive of patient prognosis. However, pre-anti-VEGF CEST MRI alone was not predictive of prognosis in these studies [52, 53].

Lesions with favorable diffusion MRI phenotypes were also characterized by a longer qT_2_ in our study. This is consistent with the notion that T_2_ relaxation time is longer in voxels with a higher water content and that T_2_ relaxation time is directly proportional to rotational movements of water molecules. Therefore, it is expected that lesions with higher ADC_L_ values, due to lower cell density and overall higher water content, will likely show longer qT_2_. This observation is consistent with previous evidence showing strong positive correlations between ADC values and qT_2_ values in gliomas [54, 55]. In our study, qT_2_ measurements were mainly included to tease out T_2_ effects from MTR_asym_ @ 3ppm measurements, rather than as an MRI metric of interest per se. Of note, qT_2_ measurements are not generally obtained with a standardized brain tumor imaging protocol [30] (contrarily to ADC and rCBV), and ADC and qT_2_ measurements are correlated and therefore redundant to some degree. Hence, using qT_2_ estimates to predict anti-VEGF treatment outcomes may be impractical, at least in retrospective studies. Conversely, a composite ADC_L_ - rCBV biomarker would be potentially more readily obtainable and testable as a way to select patients eligible for anti-VEGF therapies. If desired, estimates of qT_2_ (“effective” T_2_) can be obtained with commercial dual-echo spin-echo T_2_-weighted sequences, which is clinically-feasible as they can be acquired with no time cost compared to a single-echo spin-echo T_2_-weighted images, as discussed in a dedicated study [56]. The acquisition of a dual-echo spin-echo T_2_-weighted sequence instead of single-echo T_2_-weighted is considered a feasible variation of glioma BTIPs [29, 30]. As for the calculation of MTR_asym_ @ 3ppm from amine CEST data, it is worth noting that pulse sequence parameters can impact its calculation [57], and that multicenter datasets to validate the reproducibility of its values across institutions are currently lacking.

From a biological perspective, our results confirmed that distinct diffusion MRI phenotypes of post-chemoradiation glioblastoma, which are known to be predictive of anti-VEGF efficacy [7, 8, 10], are associated with other advanced imaging features, reflecting two distinct biological groups of lesions. From a patient selection perspective, a k-means clustering analysis showed that lesion clusters distinguished with ADC_L_, rCBV, and MTR_asym_ @ 3ppm overlap almost perfectly with the previously defined diffusion MRI phenotypes. This not only serves as a further confirmation of the validity of the ADC_L_ ≥1240 µm^2^/s threshold, but also suggests that ADC_L_ may be sufficient for patient selection in a clinical practice scenario. Nevertheless, future studies may explore a combined use of ADC_L_ and rCBV to stratify the prognosis of patients receiving anti-VEGF, and test whether it has added value compared to ADC_L_ ≥1240 µm^2^/s alone. Additionally, future studies may employ other imaging modalities to further characterize the biology of these glioblastoma subgroups, and to evaluate their potential predictive value for anti-VEGF therapies.

Our analyses did not show significant differences in sex category prevalence, MGMT status, or EGFR status between MR diffusion phenotypes. Overall, our analyses on tumor locations, using both frequency maps and location categorizations, did not reveal a convincing preferential location for high-ADC_L_ tumors compared to low-ADC_L_ tumors, or vice versa.

This study had some limitations. First, this study included post-chemoradiation glioblastomas, and not exclusively “recurrent” glioblastomas for which a clear tumor size re-growth was documented. However, it is thought that ADC_L_ phenotypes may arise after chemoradiation, possibly before tumor re-growth, and excluding lesions showing a clear growth would remarkably reduce the sample size of the study. Second, some patients received targeted therapy or immunotherapy, which may represent a confounding variable when assessing imaging features. Finally, it was not possible to test a survival stratification of the patients in this cohort according to the proposed imaging metrics, because these patients received heterogeneous treatments after the MRI timepoint, and because not all these patients had available survival data. However, the predictive value of ADC_L_ in stratifying survival under anti-VEGF treatment was extensively demonstrated in previous literature, on multiple independent cohorts. Future studies comparing homogeneous anti-VEGF and control treatment arms may assess whether other imaging metrics such as rCBV and MTR_asym_ @ 3ppm may be independent predictors of anti-VEGF success.

## Conclusions

Glioblastomas that received chemoradiation can be grouped in two groups with distinct biological features. The group with a diffusion MRI phenotype that is known to predict a favorable response to anti-VEGF (high-ADC_L_, with ADC_L_ ≥1240 µm^2^/s) also shows lower rCBV, higher MTR_asym_ @ 3ppm, and longer T_2_ relaxation times (qT_2_), compared to lesions with an unfavorable diffusion MRI phenotype (low-ADC_L_, with ADC_L_ <1240 µm^2^/s).

## Electronic supplementary material

Below is the link to the electronic supplementary material.

Supplementary Material 1

## Data Availability

Data from this cohort is available from the authors upon request.

## Abbreviations

ADC: Apparent diffusion coefficient
ADC_L_: Lowest peak of the double gaussian distribution of ADC values
CE-T_1_: T_1_-weighted images after contrast agent
CEST: Chemical exchange saturation transfer
deltaT_1_: T_1_w subtraction maps
DSC: Dynamic susceptibility contrast
EGFR: Endothelial growth factor receptor
EPI: Echo-planar imaging
DWI: Diffusion-weighted imaging
IDH: Isocitrate dehydrogenase
MGMT: O^6^-Methylguanine DNA Methyltransferase
MRI: Magnetic resonance imaging
MTR_asym_ @ 3ppm: Magnetic transfer ratio asymmetry at 3 ppm
OS: Overall survival
qT_2_: Quantitative T_2_ relaxation time
qT_2_*: Quantitative T_2_* relaxation time
rCBV: Relative cerebral blood volume
SAGE-EPI: Spin-and-gradient-echo EPI
VEGF: Vascular endothelial growth factor
T_1_w: T_1_-weighted images before contrast agent
