# Supplementary material for: Advanced imaging characterization of post-chemoradiation glioblastoma stratified by diffusion MRI phenotypes known to predict favorable anti-VEGF response
Source: J Neurooncol. 2025 Apr 14;173(3):583–94. doi: 10.1007/s11060-025-05019-8 (PMC12170782; doi:10.1007/s11060-025-05019-8)
Supplement: Supplementary file 1 — Supplementary Material 1 [file 11060_2025_5019_MOESM1_ESM.pdf]

Advanced imaging characterization of post-chemoradiation glioblastoma stratified by diffusion MRI phenotypes known to predict favorable anti-VEGF response

Supplementary Material

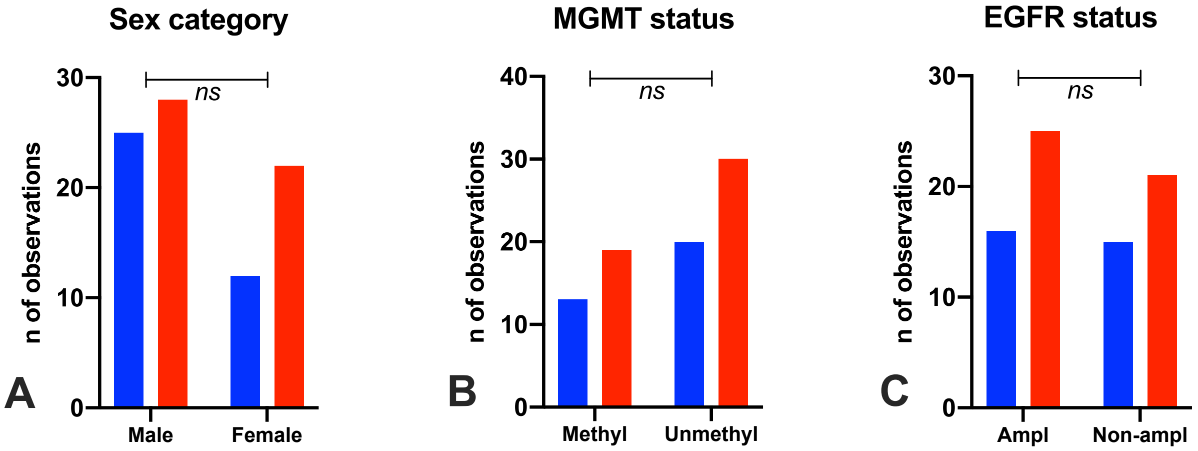

Supplementary Figure 1. Association between diffusion MRI phenotypes and clinical/pathological variables. No significant differences in the prevalence of sex category (A), MGMT methylation (B), and EGFR amplification (C) were seen between high-ADC_L (blue) and low-ADC_L (red) groups.

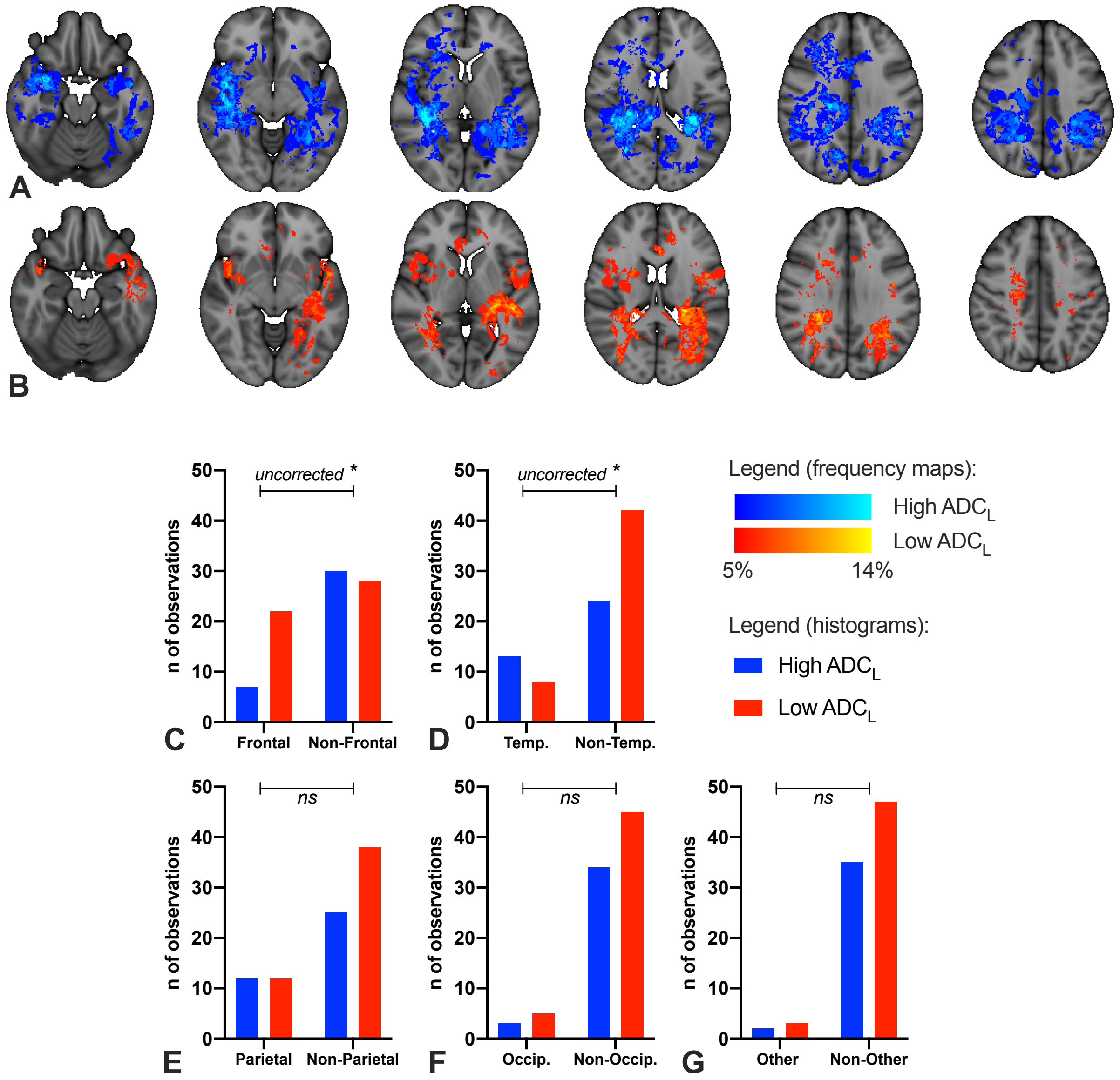

Supplementary Figure 2. Associations between diffusion MRI phenotypes and tumor locations. The frequency maps show the prevalence of high-ADC$_L$ (A) and low-ADC$_L$ lesions (B) in each voxel. When categorizing lesion locations, high-ADC$_L$ tended to be relatively less prevalent in the frontal lobe (C) and more prevalent in the temporal lobe (D), compared to low-ADC$_L$ lesions, but the statistical significance of this analysis was not confirmed after a Benjamini-Hochberg correction.

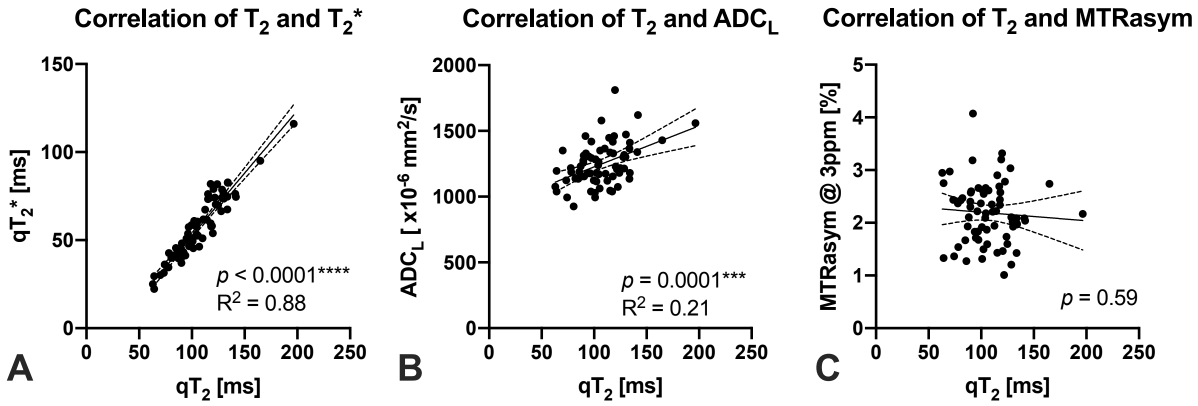

Supplementary Figure 3. Correlations between MRI quantitative metrics. Median T_2 and T_2* relaxation times extracted from the contrast-enhancing tumor are strongly correlated (A). ADC_L values and median T_2 relaxation times are moderately correlated (B). Median T_2 relaxation times and median MTR_{asym} @ 3 ppm are not significantly correlated (C).
